# Topics of Public Interest

**Published:** 1907-06

**Authors:** 


### TOPICS OF PUBLIC INTEREST.

### What to Eat.

### Some People Need Meat, but Most People Eat Too Much of It.

[*New York Tribune, May 12, 1907.*]

RECENTLY Professor Irving Fisher, of Yale University, made a series of endurance tests on forty-nine persons, representing two contrasted types of dietetic habits. It was generally reported in the press that as a result of these experiments it had been shown that vegetarians had much greater endurance than flesh eaters. This was seized upon as a victory for vegetarianism. Professor Fisher has more recently given an account of the experiments and the results, in which he suggests that the distinction should be made between high and low proteid dietaries rather than between those of vegetables and flesh. A vegetable diet is not necessarily a low proteid diet, as there are vegetables which are rich in proteids. The report of the investigation also gave no evidence that a strictly vegetarian diet was necessary in order to reach the maximum endurance. The experiments suggested that a diet of low proteid character resulted in a greater degree of endurance. Foodstuffs are divided into heat producers and tissue builders, the proteids constituting the latter.

The experiment was a part of a series of studies Professor Fisher is making on the subject of endurance from the point of view of an economist, seeking to discover what conditions favor the labor power of nations. In his studies he has pointed out that strength and endurance are not the same, the strength of a muscle being measured by the utmost force it can exert once. while its endurance is measured by the number of times it can repeat a given exertion within its strength.

The subjects of the experiment were of three classes—athletes accustomed to a high proteid and full flesh dietary, athletes accustomed to a low proteid and non-flesh dietary and sedentary persons, who lived upon food of a low proteid and non-flesh character. They consisted of Yale instructors and students, a Connecticut physician, and some of the physicians, nurses and employes of a well known vegetarian sanatorium. The personnel of the sanatorium was selected as representing abstainers from flesh foods. All of these subjects except one had abstained from meat for periods of from four to twenty years, and five of them had never eaten such food. The exception had abstained for two years only.

Two comparisons were planned, one between flesh eating athletes and flesh abstaining athletes and the other between flesh eating athletes and flesh abstaining sedentary workers. The pitting of the sedentary workers against the athletes was a handicap placed upon the "abstainers" intentionally, in order to give them a more severe and decisive test in case the first comparison between the picked athletes of both classes should turn out in their favor.

Three simple endurance tests were employed. First, the arms were held horizontally as long as possible. The second was deep knee bending and the third leg raising with the subject lying on his back. The tests were made before witnesses.

In the arm holding contest the flesh abstainers showed great superiority. Even the maximum record of the lovers of the flesh-pots was barely more than half the average for the abstainers. Only two of the fifteen flesh eaters succeeded in holding their arms out over a quarter of an hour, while twenty-two of the thirty-two of the other class surpassed that limit. None of the flesh eaters reached half an hour, but fifteen of the thirty-two abstainers exceeded that limit. Nine of them exceeded an hour; four, two hours, and one, three hours.

In respect to deep knee bending only one of the nine flesh lovers reached 1,000, as against six of the twenty-one abstainers. None of the former surpassed 2,000, as against two of the latter.

The records for leg raising showed little difference. None of the contestants reached their absolute limits. The highest record for the abstainers was 1,000 times.

The average records for each of the three classes were as follows:

Arm holding, athletic flesh eaters, 16 minutes; flesh abstainers, 31 minutes; sedentary abstainers, 62 minutes.

Deep knee bending—athletic flesh eaters, 576 times; flesh abstainers, 933 times, and sedentary abstainers, 535 times.

Professor Fisher shows why these differences could not be due to exercise, sleep or leisure on the part of the abstainers, physique or fresh air. He concludes, therefore, that the difference in endurance must be due to the diet. From examination he found that the contrast in the groups in respect to flesh was closely associated with the contrast in respect to the amount of proteid in the subjects. This examination showed that the diet of the latter was low in proteid as compared with the ordinary American diet.

Regarding the experiment Professor Fisher says:

"Of the three groups compared, the large flesh eaters showed far less endurance than the abstainers, even when the latter were leading a sedentary life. With stronger reason must the large flesh eaters of the sedentary type be inferior in endurance to abstainers. In view of the great superiority shown, the heavy handicap imposed upon the abstainers and the absence of known factors to account for their superiority, it is improbable that this superiority can be explained away by adventitious circumstances. It is possible that the superiority of the abstainers is due to the absence of flesh foods or to the use of a smaller amount of proteid, or to both, as well as to the abstention from tea, coffee and condiments.

"No attempt has been made to explain why the use of high proteid or flesh food should diminish endurance. A number of theories have been proposed. Dr. Alexander Haig, of London, has long maintained that abstainers have greater endurance than flesh eaters, particularly if they abstain also from other 'uric acid producing foods,' such as (he says) eggs, beans, peas, asparagus and mushrooms. His principal theory is that uric acid is the factor in diet which induces fatigue. Many of his theories, both as to uric acid and as to the requirements of proteid, have been overthrown, but his claims for the advantages of a purin-free—or, at any rate, a fleshless—dietary have received much corroboration. The manner in which, according to Haig, uric acid interferes with endurance is by making the blood 'collemic' or viscous, whereby it becomes difficult for the heart to pump it through the capillaries. Hence the blood pressure increases. Observations actually show that persons possessing great endurance often have low blood pressure. This is true, for instance, among the subjects of the present experiment at the sanatorium.

"A more general theory than that of Haig is that flesh foods contain in themselves 'fatigue poisons' of various kinds, which naturally aggravate the action of the fatigue poisons produced in the body.

"Still another theory in favor of the use of low proteid is that mentioned by Professor Chittenden in his 'Physiological

Economy in Nutrition,' and concerns the metabolism of proteid. As is well known, fat and carbohydrate, when consumed, give off merely carbonic acid gas and water, both of which are easily eliminated, the one being a gas and the other a liquid. Proteid, on the other hand, produces crystalline waste products, of which uric acid is one. The theory is that these midway products of metabolism in some manner produce fatigue. When the problem of the physiology of fatigue and its relations to food ingested is more fully solved, it will not only satisfy a legitimate scientific curiosity, but will point unmistakably to the optimum diet under various conditions. In the mean time it may be said that, whatever the explanation, there is strong evidence that a low proteid, non-flesh dietary is conducive to endurance.

"The truth of this result has been long obscured through two unfortunate circumstances. One is the vegetarian fanaticism, mentioned by Caspari, which has done much to defeat its own ends. From the premise, often bolstered up by theological dogma, that flesh eating is wrong, the inference is drawn that it must be unhygenic. This reasoning is so utterly at variance with the methods of modern science as to stamp those who use it as victims of bigoted prejudice, and to prevent any genuine scientific investigation. At present the tendency of such investigations as those of Chittenden, Mendel, Folin, Metchnikoff, Caspari, Le Fevre, Fauvel and others have a distinct trend toward a fleshless dietary. And yet such are the associations of the term 'vegetarian' that many are loath to grant even what is due to the tenets of 'vegetarianism.' The proper scientific attitude is to study the question of meat eating in precisely the same manner as one would study the question of bread eating.

"The second circumstance which has obscured the merits of a low proteid and non-flesh diet is that many of those who have attempted experimentally to give up flesh foods have made themselves ill. The reason formerly given for this effect was 'deficiency of proteid,' but in view of the researches of Pawlow and other recent writers we may be fairly certain that such failures are usually due to the fact that meat is a highly peptogenic food. It follows that when it is suddenly or forcibly cut out of one's diet the stomach feels the lack of its accustomed stimulus. There seem to be practical advantages in the method of reaching a low proteid diet adopted by Mr. Fletcher, consisting of thorough mastication. The experiment last year at Yale, as well as other experiments, have shown that adherence to this practice leads to a gradual reduction in proteid and flesh foods without any unpleasant or dangerous self-denial on the part of the experimenter. It may well be that those who, in spite of thorough mastication, still have a craving for flesh foods, have an actual physiological need which no other foods—at least in the list of foods employed by them—is able to satisfy. The question of the extent to which flesh foods may be used advantageously is still open, but there can now be little question, in view of the facts which have come to light during the last few years, that the ordinary consumption of those foods is excessive."

---

A Method of Reducing Old Colles's Fractures: Stiffness Following Apparent Contusions of the Elbow Joint.—Clarence A. McWilliams considers the possibility of improving the position of the fragments in cases of Colles's fractures which have gone for some time unrecognised, and have become consolidated in bad position. In some cases disability is slight and it is better to let things alone. In others it seems necessary to do something to improve matters. The author uses for refracturing the bones a large engineer's monkey wrench with the blades well padded. This is used to immobilize the upper fragment, resting against the projecting edge of the lower fragment. Thus a fracture in the line of the original injury may be produced with the expenditure of very little force. There is no strain on the structures of the wrist, and the injury to the tendons is very slight. Traumata at the elbow joint, which are treated for sprains sometimes develop stiffness later, on account of the formation of new bone within the joint, as a result of a fragment of periosteum getting into the joint and producing bone, which is so placed as to be between the ends of the bones. Usually forced persistent passive motions will crowd away the fragment of bone to the side of the joint so that it does not interfere with motion.—*Medical Record,* May 25, 1907.

---

Treatment of Fractures.—In the 1907 edition of his well-known Surgical Year Book (*Practical Medicine Series*), Prof. J. B. Murphy has the following to say concerning fractures and their treatment:

"Fractures have been too much neglected in the modern surgical amphitheater and text-books, and the percentage of bad results is proportionately increasing; imperfect reduction, improperly applied splints and casts, and prolonged immobilisation still produce their pernicious sequelæ."—*Railway Surg. Jour.*

---

Use of Pilocarpine for the Relief of Pruritus Vulvae.—John J. Reid says that the value of pilocarpine in the treatment of pruritus vulvæ and other forms of this affection has not received the recognition it deserves. The pilocarpine is to be used for this purpose in doses of from one-eighth to one-quarter grain.—*Medical Record,* May 25, 1907.

